# A quantitative survey of consumer perceptions of smart food packaging in China

**DOI:** 10.1002/fsn3.1563

**Published:** 2020-07-07

**Authors:** Ting Li, Kayna Lloyd, John Birch, Xiaomeng Wu, Miranda Mirosa, Xiaojun Liao

**Affiliations:** ^1^ College of Food Science and Nutritional Engineering China Agricultural University Beijing China; ^2^ Key Lab of Fruit and Vegetable Processing National Engineering Research Center for Fruit and Vegetable Processing Ministry of Agriculture Beijing China; ^3^ Department of Food Science University of Otago Dunedin New Zealand; ^4^ New Zealand Food Safety Science Research Centre & New Zealand China Food Protection Network Palmerston North New Zealand

**Keywords:** active packaging, consumer acceptance, consumer preference, intelligent packaging, smart packaging

## Abstract

This study quantified the acceptability of smart food packaging technologies and determined their associations with sociodemographic, attitudinal, and behavioral characteristics of consumers in China. Two quantitative surveys were conducted using an intercept method in Beijing with one for intelligent food packaging and the other for active food packaging. Chi‐square tests of independence and contingency tables were used to determine the acceptability of smart food packaging and significant associations with multiple variables. Smart packaging was accepted by 56% of participants in both surveys. Marital status and employment status were associated with the acceptance of active packaging, while consumer interactions with current food packaging were associated with the acceptance of intelligent packaging. Acceptance of both active and intelligent packaging was associated with trust in multiple institutions. This study is the first to provide broad information about Chinese consumers' acceptance of smart packaging technologies for food products. Findings from this research can contribute to further detailed consumer studies in product‐specific packaging designs.

## Graphical abstract

Marital status and employment status are associated with the acceptance of active packaging. Consumer interactions with current food packaging are associated with the acceptance of intelligent packaging. Acceptance of active packaging was associated with trust in the pharmaceutical industry, government agencies, and consumer organizations. Acceptance of intelligent packaging was associated with trust in the agricultural industry, government agencies, and consumer organizations.

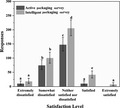

## 1 INTRODUCTION

Food safety, the assurance that a food product will not cause harm if ingested according to its intended use, is a global issue that affects consumer health in industrialized and developing countries (Lam, Remais, Fung, Xu, & Sun, 2013). For this reason, improving food safety is an ongoing and critical exploration for governments worldwide, especially as consumer concerns are rapidly increasing. Of interest for this study are the food safety concerns from Chinese individuals, as reports indicate that concerns are at an all‐time high (Ortega, Wang, Wu, & Olynk, 2011). Meanwhile, consumers are demanding better quality and taste, with concepts such as “clean label” and “minimal processing” gaining more popularity. The traditional method to ensure food safety, such as adding preservative and thermal processing, would not satisfy the need of the modern consumer. Therefore, an alternative method of assuring consumers of the safety of a food item is through effective food packaging systems. As a pervasive element involved in modern consumption behaviors, packaging provides a wide range of functionalities and benefits to consumers. Food items often rely on packaging elements to maintain product quality, prevent product losses, facilitate transportation and storage, and provide marketplace differentiation (Steenis, van Herpen, van der Lans, Ligthart, & van Trijp, 2017). While inert or traditional food packaging has provided protection for food items, complexities in distribution and consumer demand have resulted in an extensive exploration into novel food packaging techniques. These packaging techniques, often classified as “smart packaging,” encompass both active and intelligent packaging technologies (Vanderroost, Ragaert, Devlieghere, & De Meulenaer, 2014). Active packaging (AP) involves the interaction between the product, the package, and the environment (Biji, Ravishankar, Mohan, & Srinivasa Gopal, 2015; Dobrucka & Przekop, 2019). It aims to extend shelf life, maintain nutritional and organoleptic quality, inhibit pathogenic and spoilage microorganism growth, and prevent the migration of contaminants (Altan, Aytac, & Uyar, 2018; Guo, Jin, Wang, Scullen, & Sommers, 2014; Sohail, Sun, & Zhu, 2018). The primary methods of action for AP involve the absorption of oxygen, ethylene, moisture, carbon dioxide, and odors; and the release of carbon dioxide, ethanol, flavor, and antimicrobial agents (Alvarado et al., 2018; Lloyd, Mirosa, & Birch, 2019; Vermeiren, Devlieghere, van Beest, de Kruijf, & Debevere, 1999; Yildirim et al., 2018). Intelligent packaging (IP) systems aim to detect, record, trace, or communicate information regarding the product state and quality within the food chain through sensors, indicators, or radio frequency identification systems. Information concerning the origin, composition, storage condition, headspace composition, and microbial growth are all involved in IP (Aday & Yener, 2015; Lloyd et al., 2019; Realini & Marcos, 2014; Robertson, 2012; Yam, 2012).

Experts have forecasted that smart packaging is the future of food packaging (Aday & Yener, 2015; Realini & Marcos, 2014; Vanderroost et al., 2014). It is uncontested that, from a scientific standpoint, smart packaging technologies can provide a competitive advantage to products in the food distribution system (Yam, 2012). However, there are still some factors hindering the application of such technologies to food products, such as full contact material compliance, environmental sustainability, and especially perception and acceptance of consumers which is crucial to leading to success or widespread failure (O’ Callaghan & Kerry, 2016). Therefore, consumer reactions toward application of new technologies need to be taken into account before introduction.

Some smart packaging researches based in Western societies have been conducted by Aday and Yener (2015), O’Callaghan and Kerry (2016), and Barska and Joanna (2016), whereas few studies have considered consumer perception and acceptance of these novel developments in China. Previous researches conducted by Aday and Yener (2015), and Barska and Joanna (2016) indicated that education, gender, age, and brand preference influenced consumer acceptance of smart packaging technologies. Similarity, AkbayTiryaki and Gul (2007) exploring the vital factors connected with food consumption behavior in Turkey and indicated the age, income, education, household size, presence of children, and health concern had a pronounced influence on food consumption behavior (Akbay et al., 2007). Liu and Niyongira (2017) found that women, highly educated consumers, families with children, and elderly members of society have a higher level of food safety concern when compared to the rest of the population in China. And other studies about consumer acceptance of genetically modified foods, such as research conducted by Grimsrud, McCluskey, Loureiro, and Wahl (2004), found that socioeconomic characteristics were significant, with positive attitudes toward genetically modified foods linked to the young. As stated by Sajdakowska et al. (2018), younger and well‐educated people with higher income were the most innovative and unmarried respondents were more likely to accept innovations in food than those who were either or had been married in Poland. As a result, demographic variables may have an association with the acceptance of smart food packaging. Besides, consumption behavior and trust in institutions may be associated with acceptance of new technology. Graham and Jeffery (2012) indicated consumption behaviors also have impact on purchasing decision making by eye‐tracking experiment. Furthermore, a multitude of studies have shown that trust levels strongly influence purchasing decisions (Eiser, Miles, & Frewer, 2002; Groothuis & Miller, 1997; Jia & James Harvey, 2018). And a positive attitude toward trust can enhance consumer preference to technology‐embedded food (Ricci, Banterle, & Stranieri, 2018). Formulating and developing trust is complicated as it is based upon various inter‐related and nonrelated elements such as perceived accuracy, expertise, knowledge, transparency, and public concern (Peters, Covello, & McCallum, 1997). Through the use of symbolic and functional attributes, consumers construct and associate their perception and trust of a food product (Sirgy & Samli, 1985). Vandermoere, Blanchemanche, Bieberstein, Marette, and Roosen (2011) have stated that acceptance of nanotechnology in food industry is associated with consumers trust. Therefore, the questionnaire was designed based on these studies.

The investigation in this study was carried out to assess consumers' attitudes toward existing food packaging and explore factors linked with their perception and acceptance of smart packaging. In order to ensure that participants have already interacted with these items, research was conducted within an educational and relatively affluent area within Beijing.

## 2 MATERIAL AND METHODS

### 2.1 Literature research and prestudy

Due to the limited research on consumer acceptance, behavior, and knowledge regarding smart food packaging technologies, the quantitative research was preceded by intensive literature research and a qualitative prestudy to attain a well‐founded basis for the quantitative survey. Previous research has illustrated the commonality of supplementing focus groups with quantitative research, which is generally required to obtain the information needed to make substantive conclusions (Manstan & McSweeney, 2020). This manner of approach has been employed in previous research exploring packaging elements and further studies on consumer perceptions toward novel technology (Greenbaum, 1998; Huang, Qiu, Bai, & Pray, 2006; Lindh, Olsson, & Williams, 2016).

First, 16 packaging experts were interviewed individually on the topic of smart food packaging, consumer demand, and consumer acceptance of smart food packaging. The experts comprised of academics, scientists, specialist advisors, private researchers, and industry stakeholders. The goal was to obtain an understanding of the key developments in food packaging and expert opinion on consumer acceptance of these novel technologies. Secondly, five focus groups (*n* = 32) were conducted in China to determine consumer perception and acceptance of smart food packaging technologies. Data collected from focus groups are particularly sensitive to cultural variables and have accordingly been used in multiple cross‐cultural studies (Dolgopolova, Teuber, & Bruschi, 2015; Ger & Belk, 1996; Lazear, Pires, Isaacs, Chaulk, & Huang, 2008; Perrea, Grunert, & Krystallis, 2015). The semi‐structured focus groups provided an exploration into current behaviors related to food packaging and purchasing, and exploration into levels of acceptance of smart food packaging. Thus, expert opinions and consumer perception could be compared and investigated in terms of differences, knowledge gaps, and misconception. The quantitative survey reported in this manuscript was developed based on the findings of these prestudies.

### 2.2 Design and sample

This study utilized two paper‐and‐pencil surveys carried out in Beijing, China, to obtain quantitative data on consumer acceptance of the two forms of smart packaging: AP and IP. Respondents were selected based on convenience sampling, utilizing an intercept method for recruitment, as outlined by Lavrakas (2008). This nonprobability sampling method was employed as a wide range of participants were sought on a limited budget. The intercept method was conducted in the following four types of areas in Beijing: shopping malls, convenience stores (<200 m^2^), supermarkets (>200 m^2^), and tourist sites (parks, walking areas). Participants were required to be 18 years or older, currently living in China, and be primarily or jointly responsible for food management decisions (e.g., food shopping, storage decisions, food preparation) within the household. Only surveys that met the inclusion criteria were retained for analysis.

### 2.3 Materials

The survey instrument was composed in stages, using previous prestudy findings as a foundation. Three distinct sections were developed: demographics, packaging, and trust. AP and IP were split into two surveys, as initial tests indicated that inclusion of questions relating to both technologies resulted in an extended length of time (averaging 35 min) and risked participant fatigue. The two versions of the survey contained identical sections (part 1, part 2, and part 4). A differing section (part 3) of the survey explored the acceptance of either AP or IP. Both surveys were prepared in Chinese.

### 2.4 Demographic variables

Demographic questions were developed with the aid of Hughes, Camden, and Yangchen (2016) and Chan (1999). Demographic information included the following: gender, age, marital status, province, income, education, and employment. In addition to standardized demographic information, participants were asked if they had a “qualification in the field of science and technology” and “dietary requirements” in line with previous studies on novel food technologies (Ceccoli & Hixon, 2012; Grimsrud et al., 2004; Huang et al., 2006; Hudson, Caplanova, & Novak, 2015).

### 2.5 Food packaging questions

This survey sought to find relationships between existing behaviors and acceptance/rejection of smart packaging. To explore an individual's perception, opinion statements were formulated in the first person and language such as “*I like”* and “*it is important to me”* was used to encourage answers without overthinking. Prior to the introduction of smart packaging, questions regarding existing behaviors, knowledge, and current satisfaction with food packaging were presented. This order of introduction reduced the potential for response bias.

An established scale that determined health consciousness and measured consumer sensitivity to health issues was adapted for this survey. The original scale questions were developed by Kraft and Goodell (1993) and later adapted by Jayanti and Burns (1998). This study employed two questions verbatim and updated the remaining, resulting in four questions concerning consumer behavior. The questions are presented in the first column of Table 2. Responses were measured on a five‐point Likert scale ranging from “*never”* to “*almost always.”*

Depending on the survey, an introduction to either AP or IP was presented. This included a definition of the technology and two diagrams, demonstrating the packaging's primary method of action. Lay terminology was used to define technologies. Following these definitions, participants were required to respond to the following: “*I am willing to consume products that use active/intelligent packaging.”* Responses were measured on a five‐point Likert scale ranging from “*strongly disagree”* to “*strongly agre*e.” The inclusion of this question enabled later segmentation and analysis of participants.

The participants were further asked to indicate product‐specific acceptance. *The statement “I would accept active/intelligent packaging for…”* was presented. This question allowed participants to tick any relevant answer. The options included the following: dairy; fruit and vegetables; meat; and drink products.

### 2.6 Trust in institutions

Questions regarding trust were adapted from Pliner and Hobden (1992), Siegrist (2000) and Roosen et al. (2015). The scale prompted participants to indicate trust in respective institutions, regarding food safety responsibility. Six institutions were presented for evaluation: the agricultural industry, the food industry, the science/research field, the pharmaceutical industry, government agencies, and consumer organizations. The five‐point Likert scale used to measure responses ranged from “*extremely suspicious”* to “*extremely trustworthy”*.

### 2.7 Data analysis

Data analysis was performed using SPSS software (version 25). In order to examine the association between novel packaging acceptance and Chinese consumer characteristics, a single item question *“I am willing to consume products that use active/intelligent packaging”* was included in the packaging section of the survey. Respondents that “*agreed”* or “*strongly agreed”* were grouped, and others that “*disagreed”* or “*strongly disagreed”* were grouped. All participants that responded “*neither agree nor disagree”* were omitted in further analysis. A chi‐square (*χ* ^2^) test for independence was utilized. This method of analysis was chosen as other methods, such as comparisons of means and *t* tests required normally distributed results (Kinnear, 2004). When reviewing the results from this study, it was determined that the categorical outcome assumption of *t* tests did not hold. SPSS Statistics' Exact Module was used in result examination as not all results had an expected count greater than or equal to five.

Further post hoc testing utilized contingency tables. Proof and calculations of this method of post hoc testing are outlined by Beasley and Schumacker (1995) and García‐pérez and Núñez‐antón (2003). This required adjusted residuals from previous analysis to be transformed into chi‐square values, and further calculations were then utilized to determine *p*‐values. In doing so, the variable causing the statistical significance could be identified.

## 3 RESULTS

A total of 638 surveys were completed, 251 AP surveys and 387 IP surveys. All surveys incorrectly completed were purged, resulting in 241 and 371 responses, respectively. The differing sample size for each survey was due to the method of recruitment.

### 3.1 Consumer satisfaction with existing packaging

**FIGURE 1:**
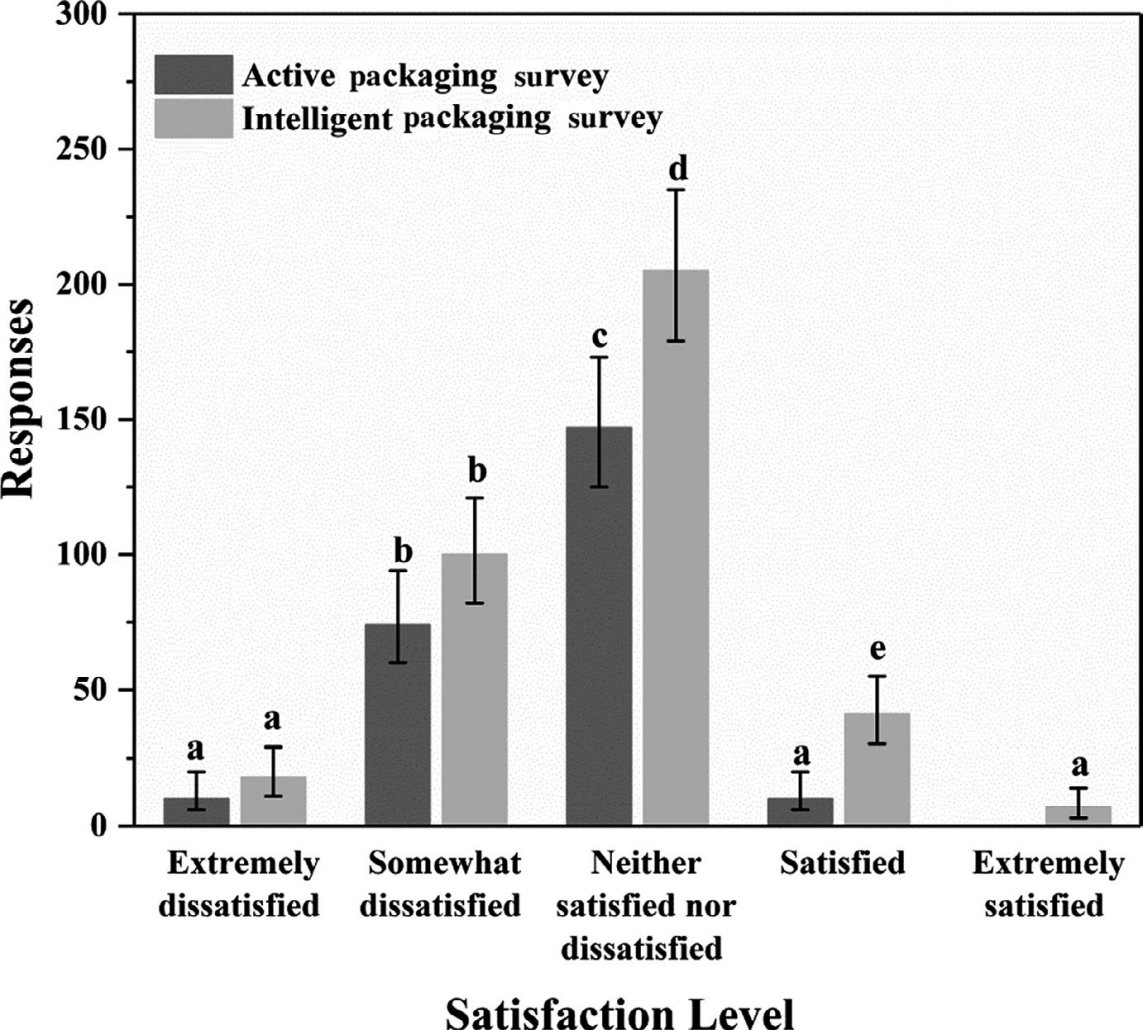
Participant satisfaction level with current food packaging. Results from active packaging survey (*n* = 241) and intelligent packaging survey (*n* = 371) with 95% confidence intervals presented

Preceding the investigation of independence and exploration into the acceptability of smart packaging, a review of consumer satisfaction with current food packaging was conducted. Figure 1 presents the findings from both surveys.

A total of 147 of the 241 AP survey respondents and 205 of the 371 IP survey respondents indicated to be “*neither satisfied nor dissatisfied”* with current packaging. These results were found to be statistically significant. Of interest, a very small number of participants indicated “*extreme satisfaction”* with current packaging.

### 3.2 Acceptance of smart packaging

The primary aim of this study was to quantify consumer acceptance levels of smart packaging. Acceptance levels were determined following the introduction of AP or IP. 56% of respondents (*n* = 135) stated AP was acceptable, 37% (*n* = 90) were undecided, and 7% (*n* = 16) deemed it to be unacceptable. Results from the IP survey found that an equal percentage of respondents, 56% (*n* = 210) stated IP was acceptable, 40% (*n* = 147) were undecided, and 4% (*n* = 14) rejected the packaging technology.

Results indicated that when participants were presented with product‐specific applications, there was increased acceptance when compared with questioning without specific application. One hundred and sixty‐four participants (68%) indicated AP was acceptable when applied to fruits and vegetables. This was followed by application in dairy (62%, *n* = 149), meat (58%, *n* = 140), and drinks (57%, *n* = 139).

These findings were mimicked in the IP survey, as 75% of participants (*n* = 279) indicated IP was acceptable when used on dairy products, followed by application in meat (73%, *n* = 270), fruit and vegetables (57%, *n* = 213), and drinks (49%, *n* = 183).

“Undecided” participants were removed from the data sets, resulting in 151 respondents from the AP survey and 224 respondents from the IP survey.

### 3.3 Demographic variables

Gender distribution was slightly skewed in both surveys with 44.4% men and 55.6% women in the AP survey and 41.1% men and 58.9% women in the IP survey. A large proportion of respondents in the AP group were between 18 and 24 (43.0%) with a cumulative percentage of 68.2% for respondents between 18 and 34. A large number of respondents were single (55.6%) and educated, with 56.3% of respondents stating to have attained a bachelor's degree. The IP group also contained a large number of respondents with tertiary education, with a cumulative percentage of 62.1%. Furthermore, results indicated that 80.8% (*n* = 181) of participants had attained a qualification in the science and technology field.

A chi‐square test for association was conducted between acceptance groups (acceptance and rejection of AP or IP) and demographic variables (gender, age, marital status, diet, income, education, employment, and science qualification). As a majority of the data violated assumption three of the chi‐square test, that all expected counts be greater than 5, exact tests were conducted to confirm significance.

Results, presented in Table 1, indicated a statistically significant association between marital status and AP acceptance, *χ* ^2^(2, *N* = 151) = 7.654, *p* = .023. There was a moderate association between these variables, *φ* = 0.225, *p* = .023. The relationship between employment and acceptance was significant, *χ* ^2^ (5, *N* = 151) = 12.219, *p* = .017, with a moderately strong association, *φ* = 0.284, *p* = .108. Post hoc testing of these significant values indicated a higher proportion of participants who were married with children found AP unacceptable. Furthermore, higher proportions of “unemployed” and “others” found AP unacceptable. Of interest, predicted associations between AP acceptance and gender, *χ* ^2^ (1, *N* = 151) = 0.342, *p* = .559, and scientific qualification, *χ* ^2^ (1, *N* = 151) = 1.673, *p* = .310 were not significant.

**TABLE 1:** Demographic variables, chi‐square test of independence

	Active packaging (*n* = 241)			Intelligent packaging (*n* = 371)		
	*χ* ^2^ ^a^		*φ* ^b^	*χ* ^2^ ^a^		*φ* ^b^
	*χ* ^2^	*p*	*φ*	*χ* ^2^	*p*	*φ*
Gender	0.342 (1)	.559	0.048	5.686 (1)	.054	0.159
Age	5.248 (4)	.265	0.186	2.336 (4)	.689	0.102
Marital status	7.654 (2)	.023*	0.225	0.867 (2)	.764	0.062
Diet	0.109 (1)	1.000	0.027	0.815 (1)	.481	0.060
Income^c^	12.700 (9)	.168	0.290	9.569 (9)	.381	0.207
Education	12.219 (6)	.108	0.284	9.358 (5)	.130	0.204
Employment	15.582 (5)	.017*	0.321	10.840 (5)	.084	0.220
Science qualification	1.673 (1)	.310	0.105	0.048 (1)	1.000	0.015

^a^ Chi‐square test of independence, *df* in parentheses.

^b^ Phi & Cramer's V Coefficient.

^c^ Monthly income (RMB).

* Significant *p* < .05

Results from the IP survey found no association between IP acceptance and demographic variables.

### 3.4 Consumer interactions with packaging

No statistically significant association was found following the chi‐square tests of association between AP acceptance and consumer behavior with current food packaging. However, there was a statistically significant association between IP acceptance and reading ingredients labels, *χ* ^2^ (4, *N* = 224) = 10.616, *p* = .033, with a moderate association, *φ* = 0.218, *p* = .033. Further significant association was found between IP acceptance and checking dates printed on packaging, *χ* ^2^ (4, *N* = 224) = 15.255, *p* = .017. This was found to be a moderately strong association, *φ* = 0.261, *p* = .017. Post hoc testing found higher proportions of participants that found IP unacceptable “never” (*p* = .0001) or “seldom” (*p* = .0013) read food labels. Furthermore, higher proportions of participants that found IP unacceptable “never” (*p* = .0003) checked dates printed on labeling (Table 2).

**TABLE 2:** Consumer interactions with packaging, chi‐square test of independence

	Active packaging (*n* = 241)			Intelligent packaging (*n* = 371)		
	*χ* ^2^ ^a^		*φ* ^b^	*χ* ^2^ ^a^		*φ* ^b^
	*χ* ^2^	*p*	*φ*	*χ* ^2^	*p*	*φ*
I usually read ingredients on food labels	2.9000 (4)	.581	0.139	10.616 (4)	.033*	0.218
I check the country‐of‐origin on food	4.676 (4)	.329	0.176	7.359 (4)	.111	0.181
I check the dates printed on products	3.626 (4)	.426	0.155	15.255 (4)	.017*	0.261
I seek information on new packaging	4.577 (4)	.334	0.174	7.803 (4)	.095	0.187

^a^ Chi‐square test of independence, *df* in parentheses.

^b^ Phi & Cramer's V Coefficient.

* Significant *p* < .05.

### 3.5 Consumer trust in various institutions

Following the chi‐square test for association, a significant relationship was found between AP acceptance and trust in the pharmaceutical industry, *χ* ^2^ (4, *N* = 151) = 11.215, *p* = .025. This relationship was determined to be moderately strong, *φ* = 0.273, *p* = .025. A moderately strong relationship, *φ* = 0.281, *p* = .024 was also found between AP acceptance and government agencies *χ* ^2^ (4, *N* = 151) = 11.902, *p* = .024. Finally, a significant relationship between AP acceptance and trust in consumer organizations was determined, *χ* ^2^ (4, *N* = 151) = 16.173, *p* = .006. There was a strong relationship between these variables, *φ* = 0.327, *p* = .006. Post hoc testing determined higher proportions of participants that found AP unacceptable was suspicious of the pharmaceutical industry (*p* = .0050), the government (*p* = .0008), and consumer organizations (*p* = .008; Table 3).

**TABLE 3:** Consumer trust levels, chi‐square test of independence

	Active Packaging (*n* = 241)			Intelligent Packaging (*n* = 371)		
	*χ* ^2^ ^a^		*φ* ^b^	*χ* ^2^ ^a^		*φ* ^b^
	*χ* ^2^	*p*	*φ*	*χ* ^2^	*p*	*φ*
Agriculture industry	2.818	.588	0.137	19.468	.003*	0.295
Food industry	5.857	.210	0.197	6.837	.128	0.175
Science and research field	9.247	.060	0.247	16.366	.066	0.271
Pharmaceutical	11.215	.025*	0.273	6.431	.167	0.169
Government agency	11.902	.024*	0.281	19.748	.019*	0.297
Consumer organizations	16.173	.006*	0.327	18.712	.002*	0.289

^a^ Chi‐square test of independence, *df* in parentheses.

^b^ Phi & Cramer's V Coefficient.

* Significant *p* < .05.

Results from the IP survey indicated moderately strong, significant relationships between acceptance of IP and trust in the agricultural industry, (*χ* ^2^ (4, *N* = 224) = 19.468, *p* = .003, *φ* = 0.295, *p* = .003), trust in government agencies (*χ* ^2^ (4, *N* = 224) = 19.748, *p* = .019, *φ* = 0.297, *p* = .0019), and trust in consumer organizations (*χ* ^2^ (4, *N* = 224) = 18.712, *p* = .002, *φ* = 0.289, *p* = .002). Participants with extreme suspicion in the agricultural industry (*p* = .0000) did not accept IP. A higher proportion of participants who were suspicious (*p* = .0026) or extremely suspicious (*p* = .0031) of government agencies indicated that IP was unacceptable. Finally, a higher ratio of participants who were suspicious of consumer organizations (*p* = .0002) indicated IP was unacceptable (Table 4).

**TABLE 4:** Post hoc contingency tabulation for significant results in active packaging survey

Variables	Level	*z*	*χ* ^2^	*p*
Marital status	Single	2.0759	4.3094	.0379***
	Married (no children)	1.1266	1.2692	.2599
	Married (with children)	−2.7055	7.3197	.0068*
Employment	Student	1.7018	2.8961	.0888
	Full‐time	0.6130	0.3758	.5399
	Part‐time	−0.6948	0.4827	.4872
	Self‐employed	0.8605	0.7405	.3895
	Unemployed	−2.5406	6.4546	.0011**
	Other	−2.5406	6.4546	.0011**
Pharmaceutical	Extremely suspicious	−1.2469	1.5548	.2124
	Suspicious	−2.7678	7.6607	.0050*
	Neutral	2.0531	4.2152	.0401***
	Trustworthy	1.1806	1.3938	.2378
	Extremely trustworthy	0.6978	0.4869	.4853
Government agency	Extremely suspicious	−0.0518	0.0027	.9587
	Suspicious	−3.3396	11.1529	.0008*
	Neutral	0.8952	0.8014	.3707
	Trustworthy	0.7886	0.6219	.4303
	Extremely trustworthy	1.1266	1.2692	.2599
Consumer organizations	Extremely suspicious	−1.5823	2.5037	.1136
	Suspicious	−3.3575	11.2728	.0008*
	Neutral	1.8923	3.5808	.0585
	Trustworthy	0.8624	0.7437	.3885
	Extremely trustworthy	1.1266	1.2692	.2599

***p < .05.

* *p* < .0083.

** *p* < .0042.

## 4 DISCUSSION

This study aimed to determine consumer acceptance of smart packaging quantifiably and to further link acceptance with behavioral, attitudinal, and demographic information. Findings from this study provide insight into prosperous areas of application for novel IP and AP technologies in China. To date, it is the first study to explore Chinese consumer acceptance of smart packaging. While generalizations were avoided, it was essential to summarize key findings that affected the acceptance of smart food packaging in China. This discussion presents findings related to consumer satisfaction with existing packaging, and variables that affect consumer acceptance of smart packaging including product‐specific application, demographics, current interactions with packaging, and consumer trust in institutions.

### 4.1 Consumer satisfaction with existing packaging

Survey results indicated that participants were, on the whole, neither satisfied nor dissatisfied with current food packaging. Remaining participants primarily indicated dissatisfaction with current food packaging. This supports findings by Ampuero and Vila (2006), Olsson and Larsson (2009), and Venter, van der Merwe, de Beer, Kempen, and Bosman (2011), wherein it was established that packaging is currently viewed as an integrated piece of a product. Consumers seem to give little consideration to a food packaging system, until the moment of disposal. While the difficulty in untangling these views has not been addressed, this finding was promising, as it highlights an opportunity for novel packaging developments, and addresses the potential of smart packaging success as reported by Aday and Yener (2015), Realini and Marcos (2014), and Vanderroost et al. (2014).

### 4.2 Acceptance of active and intelligent food packaging technologies

Survey results indicated that acceptance of AP and IP was even, with both surveys indicating 56% of participants accepted the novel packaging. However, a higher percentage of participants rejected AP. While previous studies, such as those conducted by Aday and Yener (2015), and O’ Callaghan and Kerry (2016), stated consumers had a preference for IP over AP, this study could not categorically determine whether one packaging technology was more attractive than another. When considering consumer perception and acceptance of smart packaging, it can be assumed that consumers will make a risk assessment quickly followed by a risk evaluation; that is, does the risk outweigh the benefit? However, the general lack of experience with smart packaging would have likely affected participant's abilities to evaluate the risks involved accurately. It can be assumed that as smart packaging becomes more prevalent, attitudes will become more sophisticated, with consumers making judgments on a case‐by‐case basis.

In reviewing product‐specific acceptance of AP and IP, no significant differences were detected. Previous research has suggested that consumers do not differentiate much among different applications of novel food technology but rather reject the novel application overall (Bredahl, 2001). This phenomenon was ascribed to domain‐specific, which refers to adopting innovations within specific product categories more easily and has been used in food sector (Chang Hsin, Huang Ching, Fu Chen, & Hsu Ming, 2017; Chen, 2018). Despite findings not indicating statistical significance between categories, it was determined that when presented with product‐specific applications higher percentages of respondents indicated that AP and IP were acceptable than when asked generally if AP or IP was acceptable.

### 4.3 Demographic variables

While previous research found that consumers were influenced by socioeconomic variables, scientific knowledge, and education, this study did not categorically confirm these findings. Instead, the approach presented by Zukin and Maguire (2004) was confirmed that consumption behaviors bridge economic and cultural institutions, large‐scale changes in social structure, and discourses of the self.

The results, concerning scientific background, were inconsistent with those of Ceccoli and Hixon (2012), Grimsrud et al. (2004), and Hudson et al. (2015), as no statistically significant association was found. These studies indicated that an understanding of science helped in individuals' understanding of scientific issues underlying novel food technologies and could be the corresponding reduction in uncertainty, which helps increase approval. It must be noted that these studies explored consumers' perceptions of GM foods, and comparisons with smart packaging may not be accurate. However, as stated in previous research by O’ Callaghan and Kerry (2016) in practice, consumers' knowledge and personal opinions are often separate to influences in food choice. Therefore, concluding that high levels of knowledge results in positive perceptions of novel technologies must be further verified.

When investigating consumer perceptions of GM food items, Huang et al. (2006) found that people with dietary requirements perceived GM technology differently than those without any requirements. Using these findings, it would be expected that consumers adhering to a diet would interact with a food package more and therefore may have a higher acceptance of innovative packaging. However, the results did not indicate any statistically significant interaction between the two variables.

A moderate association was determined between marital status and AP acceptance. The significant result was due to respondents married with children, as a higher ratio of participants with children found AP unacceptable. It is unsurprising, as these participants are making purchasing decisions that affect other parties. Increased caution is required by these participants when evaluating novel technologies. This finding is consistent with Liu and Niyongira (2017), who found that families with children have higher food safety concerns when compared with the rest of the Chinese population. Provision of additional information, to dissuade concerns, may be beneficial for these consumers.

Employment status and AP acceptance had a moderately strong, statistically significant association. It was determined that “unemployed” or “other” consumers were more likely to reject AP. This indicates that employed participants may have a greater appreciation for the extension of shelf life an AP solution can provide. The ready acceptance of AP by full‐time employed participants is indicatory of the consumer demand varying depending on lifestyle behaviors.

Only one demographic variable, gender, was determined to have a statistically significant association with IP. However, post hoc testing indicated that both genders were causing this significance. Previous research indicated that, generally, females had higher levels of concern for novel technology than males (Cardello, 2003; Rodríguez‐Entrena, Salazar‐Ordóñez, & Becerra‐Alonso, 2016). However, this was not affirmed by this study. Similar results have been mentioned by Sajdakowska et al. (2018) that gender has no effect on consumer opinion in some cases.

### 4.4 Current packaging interactions

It was theorized that consumers with higher levels of interaction with existing food packaging would be more accepting of novel packaging technologies. No association was determined in the AP survey. The application of AP does not increase consumer engagement, and it is therefore understandable that no association was found between interactions with current packaging and acceptance of AP. However, the IP survey determined associations with two packaging behaviors, reading ingredients on food labels, and checking dates printed on food packaging. Post hoc testing found that participants not engaging in these behaviors were less likely to accept IP. IP provides a comprehensive method to communicate with consumers. Previous studies have shown that clear communication through labeling, appearance, and design of a food package will influence the overall acceptability of a product (Ahvenainen & Hurme, 1997; Ampuero & Vila, 2006). It has been reported that a person who was used to looking for information about the country of origin was more likely to accept traceable beef steak marked by quick response (QR) code (Spence, Stancu, Elliott, & Dean, 2018), while intelligent packaging will provide more clarity and information to consumers, which will be appealing to consumers engaging with their packaging. However, if consumers are not currently seeking information from the packaging, the application of IP is not deemed necessary.

### 4.5 Consumer trust in institutions

When analyzing novel food technologies, it is common practice to explore consumers' trust in various organizations. Higher levels of trust are often associated with higher consumer confidence levels in novel food technologies (Matzembacher, Carmo Stangherlin, Slongo, & Cataldi, 2018). Results from this study indicated that low levels of trust in pharmaceutical, government, and consumer organizations affected the acceptance of AP. Furthermore, acceptance of IP was affected by low levels of trust in agriculture, government, and consumer organizations. These findings support Vandermoere et al. (2011), who stated that acceptance of novel food technologies is directly associated with consumer trust in government. Additionally, Rodríguez‐Entrena et al. (2016) confirmed that a positive association has found between trust in institution as well as science authority and acceptance to genetically modified food. Results were unexpected, as a defining feature of the Chinese population is citizenry pride, as presented by Garner (2005). However, these findings may have been influenced by reports of unsafe vaccinations shortly before conduction of the study in Beijing, reflecting findings from Peters et al. (1997), indicating trust is directly affected by current public concern.

As presented by Savadori et al. (2004) and Ricci et al. (2018), gaining consumer trust is fundamental in assuaging consumer concern, as trust minimizes uncertainty, allowing consumers to quickly and easily make decisions. When consumers know little about novel food technologies, they are strongly influenced by organizations in which they trust, impacting the perception of risk, benefit, and overall acceptance. Previous literature found that trust in institution could enhance acceptance of biotechnology and lower risk perception (Jia & James Harvey, 2018). As the results indicated, participants that were suspicious of consumer organizations were less likely to accept AP and IP. This indicated that when consumers felt safer and trusted an institution, there is a higher likelihood of gaining consumer confidence in novel packaging technology.

Understandably, Chinese consumers have high food safety concerns due to the numerous scandals that have occurred in the last decade. As stated by Xiu and Klein (2010) and Lam et al. (2013), fraudulent adulteration of products is often linked with small fragmented distribution channels. To ensure successful market uptake of smart packaging, it is crucial for steakholder to enhance confidence in food safety. As we all know, a positive attitude toward brand image or trust in institutions will lead to higher brand loyalty and better acceptance toward biotechnology, respectively (Haase, Wiedmann, & Labenz, 2018; Jia & James Harvey, 2018). Therefore, only trusted consumer organizations or brands should be used in the introduction of AP or IP.

### 4.6 Research limitations

This study has several limitations that must be acknowledged. Convenience sampling may have resulted in a sampling error and limited population representation. Due to the large sampling size, the occurrence of sampling error was significantly reduced. This sample was not representative of the Chinese population. Instead, it predicted consumer acceptance for the demographics represented. As a nonprobability sampling approach was used for this study, results could not be generalized to a larger population on statistical grounds.

## 5 CONCLUSION

This study makes an important contribution to the literature, as it is the first study to date to quantify the acceptability of smart food packaging technologies and determine associations with sociodemographic, attitudinal, and behavioral characteristics of consumers in China. Findings showed that consumer satisfaction with existing food packaging was slightly skewed to “dissatisfied.” These results indicated that there is an opportunity for improvement in food packaging. When participants were asked to indicate their acceptance of IP or AP, more than half of the respondents indicated it was acceptable. Acceptance levels increased when participants were presented with product‐specific applications, indicating that successful implementation of novel applications was reliant upon product type and category. Thus, while this study had a wide research scope, as was required due to the gap in previous academic exploration, further research with industry and product‐specific focus would be beneficial. For example, a survey on the consumer perceptions of AP in the dairy industry or an investigation of consumer attitude toward intelligent sensors on food packaging should be conducted. Studies conducted in this manner will provide rich detail and industry‐relevant results.

## CONFLICT OF INTEREST

The authors declare no financial or commercial conflict of interest.

## ETHICAL APPROVAL

This study does not involve any human or animal testing.

## INFORMED CONSENT

Written informed consent was obtained from all study participants.

## Supporting information

Appendix S1
