# Supplementary material for: A quantitative survey of consumer perceptions of smart food packaging in China
Source: Food Sci Nutr. 2020 Jul 7;8(8):3977–88. doi: 10.1002/fsn3.1563 (PMC7455960; doi:10.1002/fsn3.1563)
Supplement: Supplementary file 1 — Appendix S1 [file FSN3-8-3977-s001.docx]

**I FOOD PACKAGING SURVEY: ACTIVE PACKAGING**

PART 1:

1. What is your gender?
2. Female
3. Male
4. Other
5. Prefer not to answer
6. What is your age?
7. 17 and below
8. 18-24
9. 25-34
10. 35-44
11. 45-54
12. 55 and above
13. What is your marital status?
14. Single
15. Married, no child
16. Married with children
17. What is your monthly income (RMB)?
18. ￥3,000 and below
19. ￥3,000-￥5,999
20. ￥6,000-￥7,999
21. ￥8,000-￥9,999
22. ￥1,0000-￥14,999
23. ￥15,000-￥19,999
24. ￥20,000-￥24,999
25. ￥25,000-￥29,999
26. ￥30,000 and above
27. Prefer not to answer
28. What is the highest level of education that you have received? If currently enrolled, then highest certification received.
29. No schooling completed
30. High school
31. Diploma degree
32. Bachelor degree
33. Master degree
34. PhD
35. Prefer not to answer
36. Are you qualified in the field of science and technology?
37. No
38. Yes
39. What part of China do you come from?
40. North China
41. South China
42. East China
43. West China
44. Central China
45. Prefer not to answer
46. What is your employment status?
47. Student
48. Full time employed
49. Part time employed
50. Self-employed
51. Out of work
52. Other
53. Prefer not to answer
54. Do you have a specific diet?
55. No
56. Yes due to health problems
57. Yes due to ethical reasons
58. Yes due to sport
59. Yes due to something else

PART 2:

1. Please indicate your level of agreement with the following:

|  | 1 | 2 | 3 | 4 | 5 |
| --- | --- | --- | --- | --- | --- |
|  | Strongly  disagree | Disagree | Neither agree nor disagree | Somewhat agree | Strongly agree |
| Generally speaking, the higher the price of a product, the higher the quality |  |  |  |  |  |
| The price of a product is a good indicator of its quality |  |  |  |  |  |
| I worry that there are harmful chemicals in my foods |  |  |  |  |  |
| I am interested in information about my food |  |  |  |  |  |

1. Please rank the following from most concerned (1) to least concerned (9)

It is important to me that the food I eat on a typical day:

1. Is nutritious
2. Is easy to prepare
3. Is easily available in shops and supermarkets
4. Contains no additives
5. Contains natural ingredients
6. Is not expensive
7. Comes from countries I approve of
8. The country of origin clearly indicated
9. Is packaged in an environmentally friendly way
10. Please indicate how often do you the following:

|  | 1 | 2 | 3 | 4 | 5 |
| --- | --- | --- | --- | --- | --- |
|  | Never | Seldom | Sometimes | Often | Almost always |
| I usually read the ingredients on food labels |  |  |  |  |  |
| I check the origin of packaged products (country of origin) |  |  |  |  |  |
| I check the dates printed on packaged products |  |  |  |  |  |
| I seek out information regarding the new types of food packaging |  |  |  |  |  |

1. Overall, how satisfied or dissatisfied with the food packaging systems currently available?
2. Extremely satisfied
3. Somewhat satisfied
4. Neither satisfied nor dissatisfied
5. Somewhat dissatisfied
6. Extremely dissatisfied
   1. Please feel free to explain the reason behind your response in the space provided here.

________________________________________________________________

________________________________________________________________

________________________________________________________________

1. Would you ideally like to be provided with more information into the methods your food is packaged?
2. No
3. Yes
   1. *(If yes),* I would want this additional information to be provided:
4. Online
5. On product packaging
6. Both online and on product packaging
7. Please indicate your level of perceived importance of the following packaging features:

|  | 1 | 2 | 3 | 4 | 5 |
| --- | --- | --- | --- | --- | --- |
|  | Not important at all | Not particularly important | Neither important nor unimportant | Somewhat important | Very important |
| Product is contained and properly sealed |  |  |  |  |  |
| Pack type (metal vs plastic vs laminated paper) |  |  |  |  |  |
| Degree of decoration or appearance |  |  |  |  |  |
| Convenience features such as easy opening or big cap |  |  |  |  |  |
| Storage, stability and shelf-life of packaged product |  |  |  |  |  |
| Use of quality marks, symbols and icons – e.g. guaranteeing traceability or origin |  |  |  |  |  |
| ndication of shelf-life or freshness |  |  |  |  |  |
| Presence of tamper evidence features or tamper-proof seals and closures |  |  |  |  |  |
| Environmentally friendly aspects |  |  |  |  |  |

1. Have you heard of smart packaging for food products?
2. No
3. Yes
   1. *(If yes),* please write a short definition of what you understand the term smart packaging to mean in the space provided here.

________________________________________________________________

________________________________________________________________

________________________________________________________________

PART 3:

1. Please indicate your level of agreement with the following:

|  | 1 | 2 | 3 | 4 | 5 |
| --- | --- | --- | --- | --- | --- |
|  | Strongly disagree | Disagree | Neither agree nor disagree | Somewhat agree | Strongly agree |
| Active packaging systems are healthy |  |  |  |  |  |
| Active packaging will help keep foods fresh |  |  |  |  |  |
| Active packaging is expensive |  |  |  |  |  |
| Active packaging is safe |  |  |  |  |  |
| I am more likely to buy or consumer products that use active packaging |  |  |  |  |  |
| I am willing to buy or consume products that use active packaging |  |  |  |  |  |

1. Does your willingness to buy or consume products that use active packaging depend on the food or drink?
2. No
3. Yes
   1. *(If yes),* please explain in the space provided here.

________________________________________________________________

________________________________________________________________

________________________________________________________________

1. I would accept active packaging for *(Check all that apply)*
2. Dairy products
3. Fruit and vegetables
4. Meat products
5. Drink products
6. Does your willingness to buy or consume products that use active packaging depend on the type of smart packaging used?
7. No
8. Yes
   1. *(If yes),* please explain in the space provided here.

________________________________________________________________

________________________________________________________________

________________________________________________________________

1. Please rank the importance of these potential constraints for not purchasing active packaging products.

*Please rank the following from the most important constraint (1) to least important constraint (5)*

1. Lack of awareness
2. Not easily available
3. High price
4. Misconception about the packaging
5. Inclination towards traditional packaging

PART 4:

1. I would like my food packaging to:

*Please rank the following from the most important (1) to least important (5)*

1. Remove the need for preservative in food
2. Monitor food in the supply chain
3. Inform me if a product has been tampered with.
4. Show full product traceability
5. Extend the shelf life of the product
6. How much do you trust the following institutions in regard to their responsibility over food safety?

|  | 1 | 2 | 3 | 4 | 5 |
| --- | --- | --- | --- | --- | --- |
|  | Never | Seldom | Sometimes | Often | Almost always |
| Agriculture industry |  |  |  |  |  |
| Food-industry |  |  |  |  |  |
| Science/research field |  |  |  |  |  |
| Pharmaceutical industry |  |  |  |  |  |
| Government agency/public organisations |  |  |  |  |  |
| Consumer organisations |  |  |  |  |  |

1. Do you agree with the following statement:

“New government policy regarding food packaging will make more likely to purchase new technology.”

1. Strongly disagree
2. Somewhat disagree
3. Neither agree nor disagree
4. Somewhat agree
5. Strongly agree

**II FOOD PACKAGING SURVEY: INTELLIGENT PACKAGING**

PART 1:

What is your gender?

1. Female
2. Male
3. Other
4. Prefer not to answer

What is your age?

1. 17 and below
2. 18-24
3. 25-34
4. 35-44
5. 45-54
6. 55 and above

What is your marital status?

1. Single
2. Married, no child
3. Married with children

What is your monthly income (RMB)?

1. ￥3,000 and below
2. ￥3,000-￥5,999
3. ￥6,000-￥7,999
4. ￥8,000-￥9,999
5. ￥1,0000-￥14,999
6. ￥15,000-￥19,999
7. ￥20,000-￥24,999
8. ￥25,000-￥29,999
9. ￥30,000 and above
10. Prefer not to answer

What is the highest level of education that you have received? If currently enrolled, then highest certification received.

1. No schooling completed
2. High school
3. Diploma degree
4. Bachelor degree
5. Master degree
6. PhD
7. Prefer not to answer

Are you qualified in the field of science and technology?

1. No
2. Yes

What part of China do you come from?

1. North China
2. South China
3. East China
4. West China
5. Central China
6. Prefer not to answer

What is your employment status?

1. Student
2. Full time employed
3. Part time employed
4. Self-employed
5. Out of work
6. Other
7. Prefer not to answer

Do you have a specific diet?

1. No
2. Yes due to health problems
3. Yes due to ethical reasons
4. Yes due to sport
5. Yes due to something else

PART 2:

Please indicate your level of agreement with the following:

|  | 1 | 2 | 3 | 4 | 5 |
| --- | --- | --- | --- | --- | --- |
|  | Strongly  disagree | Disagree | Neither agree nor disagree | Somewhat agree | Strongly agree |
| Generally speaking, the higher the price of a product, the higher the quality |  |  |  |  |  |
| The price of a product is a good indicator of its quality |  |  |  |  |  |
| I worry that there are harmful chemicals in my foods |  |  |  |  |  |
| I am interested in information about my food |  |  |  |  |  |

1. Please rank the following from most concerned (1) to least concerned (9)

It is important to me that the food I eat on a typical day:

1. Is nutritious
2. Is easy to prepare
3. Is easily available in shops and supermarkets
4. Contains no additives
5. Contains natural ingredients
6. Is not expensive
7. Comes from countries I approve of
8. The country of origin clearly indicated
9. Is packaged in an environmentally friendly way
10. Please indicate how often do you the following:

|  | 1 | 2 | 3 | 4 | 5 |
| --- | --- | --- | --- | --- | --- |
|  | Never | Seldom | Sometimes | Often | Almost always |
| I usually read the ingredients on food labels |  |  |  |  |  |
| I check the origin of packaged products (country of origin) |  |  |  |  |  |
| I check the dates printed on packaged products |  |  |  |  |  |
| I seek out information regarding the new types of food packaging |  |  |  |  |  |

1. Overall, how satisfied or dissatisfied with the food packaging systems currently available?
2. Extremely satisfied
3. Somewhat satisfied
4. Neither satisfied nor dissatisfied
5. Somewhat dissatisfied
6. Extremely dissatisfied
   1. Please feel free to explain the reason behind your response in the space provided here.

________________________________________________________________

________________________________________________________________

________________________________________________________________

1. Would you ideally like to be provided with more information into the methods your food is packaged?
2. No
3. Yes
   1. *(If yes),* I would want this additional information to be provided:
4. Online
5. On product packaging
6. Both online and on product packaging
7. Please indicate your level of perceived importance of the following packaging features:

|  | 1 | 2 | 3 | 4 | 5 |
| --- | --- | --- | --- | --- | --- |
|  | Not important at all | Not particularly important | Neither important nor unimportant | Somewhat important | Very important |
| Product is contained and properly sealed |  |  |  |  |  |
| Pack type (metal vs plastic vs laminated paper) |  |  |  |  |  |
| Degree of decoration or appearance |  |  |  |  |  |
| Convenience features such as easy opening or big cap |  |  |  |  |  |
| torage, stability and shelf-life of packaged product |  |  |  |  |  |
| Use of quality marks, symbols and icons – e.g. guaranteeing traceability or origin |  |  |  |  |  |
| Indication of shelf-life or freshness |  |  |  |  |  |
| Presence of tamper evidence features or tamper-proof seals and closures |  |  |  |  |  |
| Environmentally friendly aspects |  |  |  |  |  |

1. Have you heard of smart packaging for food products?
2. No
3. Yes
   1. *(If yes),* please write a short definition of what you understand the term smart packaging to mean in the space provided here.

________________________________________________________________

________________________________________________________________

________________________________________________________________

PART 3:

1. Please indicate your level of agreement with the following:

|  | 1 | 2 | 3 | 4 | 5 |
| --- | --- | --- | --- | --- | --- |
|  | Strongly disagree | Disagree | Neither agree nor disagree | Somewhat agree | Strongly agree |
| Intelligent packaging systems are healthy |  |  |  |  |  |
| Intelligent packaging will help keep foods fresh |  |  |  |  |  |
| Intelligent packaging is expensive |  |  |  |  |  |
| Intelligent packaging will improve traceability |  |  |  |  |  |
| Intelligent packaging will stop food fraud |  |  |  |  |  |
| I am more likely to buy or consume products that use intelligent  packaging |  |  |  |  |  |
| I am willing to buy or consume products that use intelligent packaging |  |  |  |  |  |

1. Does your willingness to buy or consume products that use intelligent packaging depend on the food or drink?
2. No
3. Yes
   1. *(If yes),* please explain in the space provided here.

________________________________________________________________

________________________________________________________________

________________________________________________________________

1. I would accept intelligent packaging for *(Check all that apply)*
2. Dairy products
3. Fruit and vegetables
4. Meat products
5. Drink products
6. Does your willingness to buy or consume products that use intelligent packaging depend on the type of smart packaging used?
7. No
8. Yes
   1. *(If yes),* please explain in the space provided here.

________________________________________________________________

________________________________________________________________

________________________________________________________________

1. Please rank the importance of these potential constraints for not purchasing intelligent packaging products.

*Please rank the following from the most important constraint (1) to least important constraint (5)*

1. Lack of awareness
2. Not easily available
3. High price
4. Misconception about the packaging
5. Inclination towards traditional packaging

PART 4:

1. I would like my food packaging to:

*Please rank the following from the most important (1) to least important (5)*

1. Remove the need for preservative in food
2. Monitor food in the supply chain
3. Inform me if a product has been tampered with.
4. Show full product traceability
5. Extend the shelf life of the product
6. How much do you trust the following institutions in regard to their responsibility over food safety?

|  | 1 | 2 | 3 | 4 | 5 |
| --- | --- | --- | --- | --- | --- |
|  | Never | Seldom | Sometimes | Often | Almost always |
| Agriculture industry |  |  |  |  |  |
| Food-industry |  |  |  |  |  |
| Science/research field |  |  |  |  |  |
| Pharmaceutical industry |  |  |  |  |  |
| Government agency/public organisations |  |  |  |  |  |
| Consumer organisations |  |  |  |  |  |

1. Do you agree with the following statement:

“New government policy regarding food packaging will make more likely to purchase new technology.”

1. Strongly disagree
2. Somewhat disagree
3. Neither agree nor disagree
4. Somewhat agree
5. Strongly agree
